# The long-term evaluation of the prognosis of implants with acid-etched surfaces sandblasted with alumina: a retrospective clinical study

**DOI:** 10.1186/s40902-020-00255-6

**Published:** 2020-04-08

**Authors:** Min-Joong Kim, Pil-Young Yun, Na-Hee Chang, Young-Kyun Kim

**Affiliations:** 1 Department of Oral and Maxillofacial Surgery, Section of Dentistry, Seoul National University Bundang Hospital, 300, Gumi-dong, Bundang-gu, Seongnam-si, Gyeonggi-do 463-707 Korea; 2 Department of Biomedical Research Institute, Seoul National University Bundang Hospital, Seongnam-si, Korea; 3 Department of Dentistry & Dental Research Institute, School of Dentistry, Seoul National University, Seoul, South Korea

**Keywords:** Dental implants, Osstem implants, SA surface implants, SLA implants, Osseointegration

## Abstract

**Background:**

The aim of this study was to evaluate the long-term clinical stability of implants with acid-etched surfaces sandblasted with alumina using retrospective analyses of the survival rate, success rate, primary and secondary stability, complications, and marginal bone loss of the implants.

**Methods:**

Patients who had implants placed (TS III SA, SS II SA, SS III SA, and U III SA) with SA surfaces from Osstem (Osstem Implant Co., Busan, Korea) at the Seoul National University Bundang Hospital, from January 2008 to December 2010 were selected for the study. Patients’ medical records and radiographs (panorama, periapical view) were retrospectively analyzed to investigate sex, age, location of implantation, diameter, and length of the implants, initial and secondary stability, presence of bone grafting, types of bone grafting and membranes, early and delayed complications, marginal bone loss, and implant survival rate.

**Results:**

Ninety-six implants were placed in 45 patients. Five implants were removed during the follow-up period for a total survival rate of 94.8%. There were 14 cases of complications, including 6 cases of early complications and 8 cases of delayed complications. All five implants that failed to survive were included in the early complications. The survival of implants was significantly associated with the occurrence of complications and the absorption of bone greater than 1 mm within 1 year after prosthetic completion. In addition, the absorption of bone greater than 1 mm within 1 year after prosthetic completion was significantly associated with the occurrence of complications, primary stability, and implant placement method. Five cases that failed to survive were all included in the early complications criteria such as infection, failure of initial osseointegration, and early exposure of the fixture.

**Conclusions:**

Of the 96 cases, 5 implants failed resulting in a 94.8% survival rate. The failed implants were all cases of early complications such as infection, failure of initial osseointegration, and early exposure of the fixtures. Peri-implantitis was mostly addressed through conservative and/or surgical treatment and resulted in very low prosthetic complications. Therefore, if preventive measures are taken to minimize initial complications, the results can be very stable.

## Background

Early implants presented with mechanically polished flat surfaces, but the long-term prognosis of the rough surface of current implants has been much better in several studies. The purpose of the surface treatment of implants is (1) to increase the surface area providing greater mechanical surface fixation between the bone and the implant immediately after implant placement, (2) to provide a surface form that facilitates the maintenance of blood clots, and (3) to provide a surface shape to promote the healing process.

Several methods have been introduced that can significantly increase bone adhesion while simultaneously increasing the initial fixation force by roughening the implant surface. Representative methods include titanium plasma spray, HA (hydroxyapatite)-coating, blasting method which sprays a variety of specific particle media with excellent biocompatibility onto the implant surface, acid etching which etches on the surface of the implant with a high temperature acidic solution to increase roughness, porous sintered, and anodic oxidation.

Albrektsson et al. reported faster bone growth and better physical adhesion when the implant surface has been roughened rather than a machined surface resulting in the better adherence of osteoblasts to the implant roughened surface, thus affecting maturation, differentiation, and binding between the bone and the implant [1]. Wennerberg et al. also reported that rough surface implants had larger bone-to-implant contact and higher removal torque compared to machined surface implants [2].

The SLA method was developed by mixing the blasting method with the acid-etched method. The SLA method is known to maximize the roughness of the surface through the spraying of large particles (250–500 μm) to form macro-roughness and obtaining micro-roughness through acid corrosion (HCl/H_2_SO_4_) [3, 4].

Hak-kyun Kim et al. reported that implants with SLA surfaces have very good survival rates, and that implants with SLA surfaces appear to be particularly superior in relatively poor bone areas, such as the maxilla [5]. Buser et al. reported that the 10-year survival rate of implants with SLA surfaces was 98.8%, and the incidence of peri-implantitis over 10 years was 1.8% [6].

Osstem (Busan, Korea) has developed an SA surface implant using sandblasting with alumina and acid etching. This study is a retrospective observational study of Osstem implants with SA surfaces conducted between 2008 and 2010 and aims to evaluate the long-term clinical stability of SA surface implants by analyzing the alveolar bone height and implant survival rate after 1 year of loading and during final observation.

## Materials and methods

### Medical record analysis

Patients who had implants placed (TS III SA, SS II SA, SS III SA, and U III SA) with SA surfaces from Osstem (Osstem Implant Co., Busan, Korea) at the Seoul National University Bundang Hospital, from January 2008 to December 2010 were selected for the study. This retrospective clinical study was conducted after receiving approval from the Institutional Review Board of Seoul National University Bundang Hospital (IRB No: B-1907-555-105). Patients’ medical records and radiographs (panorama, periapical view) were retrospectively analyzed to investigate sex, age, location of implantation, diameter, and length of the implants, initial and secondary stability, presence of bone grafting, types of bone grafting and membranes, early and delayed complications, marginal bone loss, and implant survival rate.

### Stability and survival rate of the implants

**Fig. 1:**
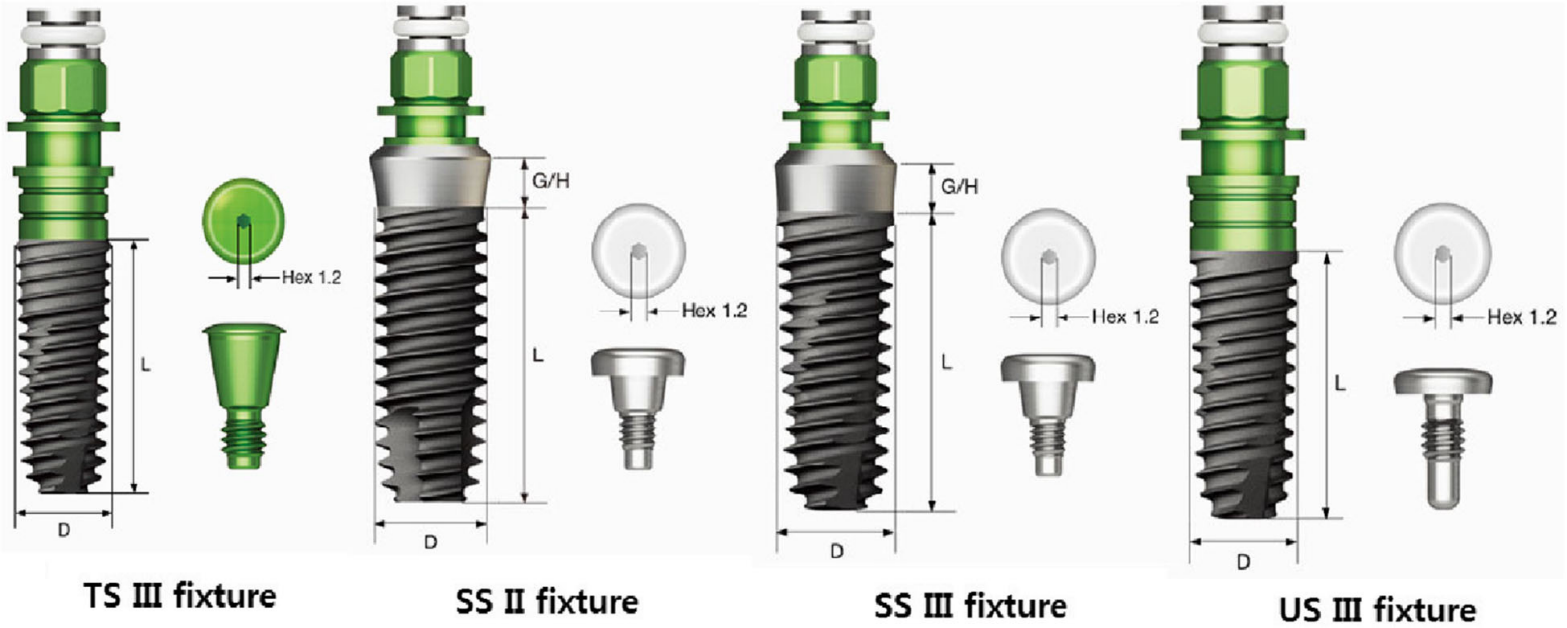
Osstem implant TS III, SS II, SS III, and Us III fixtures

The following is a schematic of Osstem’s implants, which is the subject of this study (Fig. 1).

Osstem’s TS-implant fixture is a submerged type of fixture with an internal HEX and a 11-degree Morse taper structure. By using an internal platform structure, the structural stability against external loads is high and the platform switching effect results in low bone resorption, excellent aesthetics, and placement below the bone level. Osstem’s SS implant fixture is a non-submerged type fixture with an internal Octa connection. There is an advantage that the fixture itself is a structure that penetrates the gums and does not require a secondary surgery. The structure involving the direct contact of the crown and fixture is mainly used for areas of large molar pressure due to its high structural stability against external loads. The difference between an SS II fixture and an SS III fixture is the angle of the outer surface of the fixture to the central axis of the fixture. The SS II fixture has a straight body with the depth of implantation that can be easily adjusted. It has the an advantage of being less sensitive to bone diameters or diameters of the drill. The SS III fixture is a tapered body with an angle of 1.5° and is advantageous for initial loading and early loading because of its ability for initial high stability acquisition. Osstem’s US implant fixture is a submerged type of fixture with an external hex connection. It has the advantage that the connection with the abutment is easy because the connection part with the abutment is projected outward.

Implant stability quotients (ISQ) were measured with Smartpeg™ (Osstell AB. Göteborg, Sweden) and Osstell Mentor® (Osstell, Göteborg, Sweden). Initial stability was measured immediately after implant placement, while secondary stability was measured at the time of the secondary surgery or impression with a healing abutment.

Implant survival criteria were set when the upper prosthesis was functioning normally without symptoms after installation [7].

### Marginal bone loss

**Fig. 2:**
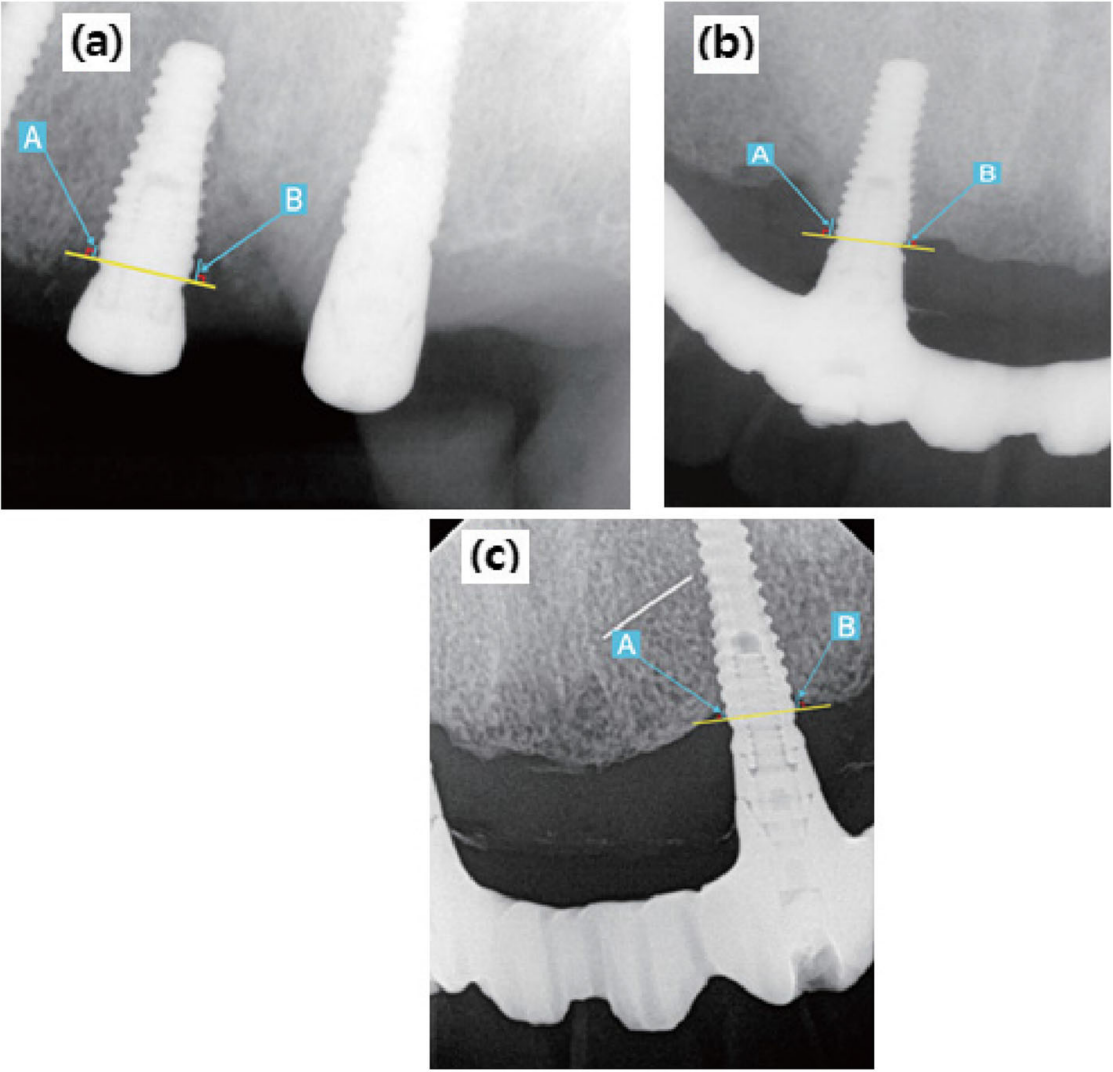
Landmarks of the radiographic measurements. **a** Immediately after surgery periapical view. **b** Periapical view 1 year after prosthetic loading. **c** Final periapical radiograph. [A point: linear distance from the implant shoulder to the contact point of the implant and bone (mesial surface), B point: linear distance from the implant shoulder to the contact point of the implant and bone (distal surface). The average value of A point and B point was set as the marginal bone loss amount.]

Radiologic analyses were performed to determine the amount of marginal bone loss according to the time of each implant placement. The evaluation of marginal bone resorption was based on radiographs taken immediately after implant placement. Using the most recent radiographs, the average height was determined by measuring the change in height from the implant to the implant-bone contact point of the implant. The radiographic linear distances from the implant shoulder to the implant-bone contact points located at the apex of the implant were measured at the mesial plane (A point of Fig. 2) and the distal plane (B point of Fig. 2). The obtained mean value was then set as the marginal bone loss amount [8].

We observed the marginal bone loss with panoramic and periapical radiographs from the time of implant placement as baseline to the last follow-up using the INFINITT PACS 3.0 (INFINITT Healthcare Co., Ltd. Seoul, Korea) software installed in Orthoceph OC100 CR (Instrumentarium Imaging, Tuusula, Finland), and Heliodent DS (Sirona, Bensheim, Germany) x-ray machine. All periapical radiographs were taken using bisecting angle technique, positioning the tube head in direction of the center of the object. Linear measurement of marginal bone resorption was obtained by one examiner, by calculating the average of mesial and distal distance from implant shoulder to alveolar crest, which were determined by multiplying the number of exposed threads and pitch distance provided by the manufacturer for each implants.

### Statistical analysis

Bivariate correlation analyses between the occurrence of complications, bone resorption within 1 year after completion of the prosthesis, the implant installation method, initial stability, presence of bone grafting, and implant survival were accomplished using IBM SPSS Statistics (Version 18; SPSS Inc., Chicago, IL, USA). Also, one-way ANOVA and independent *t* test were accomplished for comparison between the groups using IBM SPSS Statistics (Version 18; SPSS Inc., Chicago, IL, USA).

## Results

A total of 96 SA surface implants were placed in 45 patients. Fifty implants were placed in 26 male patients and 40 implants were placed in 19 female patients in this study. The average age of the patients was 59.3 years (range 28–80 years). The implants placed were 64 TS SA implants, 22 SS SA implants, and 10 US SA implants. Ten implants were placed in the anterior region, 25 in the premolar region, and 61 in the molar region. Of the 96 implants, bone grafting was performed for 76 implants, while 20 were placed without bone grafting. Of the 76 implants undergoing bone grafting, 65 were placed at the same time as the bone graft and 11 underwent delayed placement after grafting. In cases of delayed placement, the average time from bone grafting to implant placement was 4.7 months. The final survival rate of the bone-grafted implants was 94.8% with a final marginal bone uptake of 0.5 mm. The final survival rate of the implants not requiring bone grafting was 95% with a final marginal bone absorption of 0.53 mm. There was no statistically significant difference between the group with bone grafting and the group not requiring bone grafting.

Of the 96 implants, 33 implants involved one-stage surgery and 63 implants required two-stage surgery. The average healing time from the placement of the implant to the first impression or second surgery was 4 months. The average observation period was 67 months after prosthesis placement. The final survival rate for one-stage implants was 100% with a final marginal bone absorption of 0.51 mm. The final survival rate for two-stage implants was 92% with a final marginal bone absorption of 0.55 mm.

Of the 96 implants, 5 of the 96 implants were removed resulting in a survival rate of 94.8%. The causes of failure were failure of initial osseointegration, infection, and failure of the bone graft. The prostheses of 91 implants that survived at the last follow-up included 19 single fixed cases, 57 multiple fixed cases, 5 fixed hybrid cases, 5 overdenture cases, 1 removable partial denture case, and 4 dropout cases.

Complications occurred in 14 of the 96 implants, accounting for 17% of the implants. Among them, 6 early complications and 8 delayed complications occurred (Table 1).

**Table 1:** Early and delayed complications

Early complications			Delayed complications		
Type	Initial osseointegration failure	3 cases	Type	Peri-implantitis	7 cases
	Infection	2 cases		Screw loosening	1 case
	Early exposure of the fixture	1 case			

All the five implants removed had early complications. The remaining complications were resolved through treatment with peri-implant curettage, medication, and prosthesis reconstruction.

Implant stability quotients (ISQ) were measured using the Osstell Mentor (Osstell, Gothenburg, Sweden). The average initial stability was 69 ISQ and secondary stability was 74 ISQ on average. There were 16 cases of initial stability and 6 cases of secondary stability with average ISQ values of less than 60 (Table 2).

**Table 2:** Implant primary and secondary stability

Primary stability		Secondary stability	
Under 60 ISQ	16 cases	Under 60 ISQ	6 cases
60 or more ISQ	80 cases	60 or more ISQ	90 cases
Average ISQ	69	Average ISQ	74

According to the analysis of the stability difference between the implant systems, the initial stability of TS SA implants was 67 ISQ with secondary stability of 72 ISQ. The initial stability of SS SA implants was 73 ISQ with secondary stability of 76 ISQ. The initial stability of US SA implants was 77 ISQ with secondary stability 81 ISQ on average (Table 3).

**Table 3:** Clinical findings of the installed implants

	Primary stability (ISQ)		Secondary stability (ISQ)	
TS SA implant	Mean ± SD	66.6 ± 12.1	Mean ± SD	71.9 ± 11.6
	Minimum	22	Minimum	30
	Maximum	84	Maximum	88
SS SA implant	Mean ± SD	72.8 ± 10.7	Mean ± SD	75.9 ± 5.1
	Minimum	48	Minimum	64
	Maximum	90	Maximum	82
US SA implant	Mean ± SD	77.4 ± 6.7	Mean ± SD	80.6 ± 6.0
	Minimum	64	Minimum	74
	Maximum	87	Maximum	91
Average	69.1 ± 11.9		73.6 ± 10.7	

The *p* value between the TS SA implant group and the SS SA implant group was 0.077, the *p* value between the SS SA implant group and the US SA implant group was 0.537, and the *p* value between the TS SA implant group and the US SA implant group was 0.067. There was no significant difference between the other groups

The *p* value between the TS SA implant group and the SS SA implant group was 0.077, the *p* value between the SS SA implant group and the US SA implant group was 0.537, and the *p* value between the TS SA implant group and the US SA implant group was 0.067.

The primary and secondary stability of TS SA, SS SA, and US SA implants did not exhibit statistically significant differences among the groups (*p* > 0.05).

### Survival rate according to implant type

Sixty-four TS SA implants were placed in 29 patients with 62 implants (97%) surviving. Twenty-two SS SA implants were placed in 12 patients and 21 implants (95%) survived. Ten US SA implants were placed in 4 patients and 8 implants (80%) survived (Table 4).

**Table 4:** Survival rates of implants

	Survival rate (%)	Failure (*n*)
TS SA implant	62/64 (97%)	2
SS SA implant	21/22 (95%)	1
US SA implant	8/10 (80%)	2
Average	91/96 (94.8%)	Total, 5

The *p* value between the TS SA implant group and the SS SA implant group was 0.963, the *p* value between the SS SA implant group and the US SA implant group was 0.067, and the *p* value between the TS SA implant group and the US SA implant group was 0.161. There was no significant difference between the other groups

The *p* value between the TS SA implant group and the SS SA implant group was 0.963, the *p* value between the SS SA implant group and the US SA implant group was 0.067, and the *p* value between the TS SA implant group and the US SA implant group was 0.161.

The survival rates of TS SA, SS SA, and US SA implants did not exhibit statistically significant differences among the groups (*p* > 0.05).

### Marginal bone loss

The average bone loss at 1 year after completion of the prosthesis was 0.37 mm, and the average bone loss at final observation was 0.5 mm. There were 10 cases of bone loss greater than 1 mm within 1 year and all the cases involved TS SA implants. The mean marginal bone resorption after 1 year of loading and final observation in each group was 0.37 mm, 0.51 mm for TS SA implants, 0.16 mm, 0.32 mm for SS SA implants, and 0.43 mm, 0.58 mm for US SA implants, respectively. There was no significant difference between the implant system groups for marginal bone loss after 1 year of loading. As a result of analyzing the difference between each implant system for marginal bone loss at final observation, there was a statistically significant difference between the TS SA implant group and the SS SA implant group (*p* = 0.038). However, the *p* value between the SS SA implant group and the US SA implant group was 0.815, and the *p* value between the TS SA implant group and the US SA implant group was 0.575. There was no significant difference between the other groups (Table 5).

**Table 5:** Marginal bone loss (mm)

	1 year after loading	Final observation	More than 1 mm within 1 year (*n*)
TS SA implant	0.37 mm	0.51 mm	10
SS SA implant	0.16 mm	0.32 mm	0
US SA implant	0.43 mm	0.58 mm	0
Average	0.37 mm	0.5 mm	Total, 10

There was a statistically significant difference between the TS SA implant group and the SS SA implant group (*p* = 0.038). However, the *p* value between the SS SA implant group and the US SA implant group was 0.815, and the *p* value between the TS SA implant group and the US SA implant group was 0.575. There was no significant difference between the other groups

### Survival rate and marginal bone loss according to implant diameter

One case failed at 3.5 mm in diameter, two cases at 4.0 mm in diameter, and one case at 4.5 mm in diameter. The average final marginal bone loss was 4.0 mm > 3.5 mm > 4.5 mm > 5.0 mm > 4.1 mm > 4.8 mm, but there was no statistically significant difference among the groups (*p* = 0.244) (Table 6).

**Table 6:** Survival rate and marginal bone loss according to fixture diameter

Diameter	Survival rate	Marginal bone loss (1 year)	Marginal bone loss (final observation)
3.5 mm	2/3 (67%)	0.68 mm	0.55 mm
4.0 mm	23/25 (92%)	0.46 mm	0.63 mm
4.1 mm	7/7 (100%)	0.17 mm	0.25 mm
4.5 mm	12/13 (92%)	0.40 mm	0.53 mm
4.8 mm	8/8 (100%)	0.13 mm	0.20 mm
5.0 mm	39/40 (98%)	0.35 mm	0.51 mm

There was no statistically significant difference among the groups. (*p* = 0.244)

### Survival rate and marginal bone loss according to implant length

Survival failures were found in 1 case 7.0 mm in length, 2 cases 10.0 mm in length, and 2 cases 13.0 mm in length. The final marginal bone loss was 8.5 mm > 13.0 mm > 10.0 mm, 11.5 mm > 7.0 mm, but there was no statistically significant difference between the groups (*p* = 0.185) (Table 7).

**Table 7:** Survival rate and marginal bone loss according to fixture length

Length	Survival rate	Marginal bone loss (1 year)	Marginal bone loss (final observation)
7.0 mm	14/15 (93%)	0.34 mm	0.5 mm
8.5 mm	9/9 (100%)	0.44 mm	0.64 mm
10.0 mm	37/39 (95%)	0.36 mm	0.51 mm
11.5 mm	23/23 (100%)	0.34 mm	0.51 mm
13.0 mm	8/10 (80%)	0.4 mm	0.58 mm

There was no statistically significant difference between the groups. (*p* = 0.185)

### Analysis of factors that influenced implant failure (Table 8)

There were 5 cases of implants that failed to survive during the observation period. There was a significant correlation (*p* < 0.05) between survival and complications, and more than 1 mm of bone loss within 1 year after completion of the prosthesis. There was no statistically significant correlation between initial stability less than 60, the presence of bone grafting, and the implant placement method (*p* > 0.05). Bone loss of 1 mm or more within 1 year after prosthesis completion was significantly correlated with the incidence of complications, initial stability less than 60, and implant placement method (*p* < 0.05). In other words, more than 1 mm of bone resorption occurred within 1 year after prosthesis completion when complications occurred, initial stability was less than 60, or implant placement involved a 2-stage process. In addition, the implant placement method had a significant correlation (*p* < 0.05) with the incidence of complications, and the incidence of complications increased when the implants were placed in a 2-stage process.

**Table 8:** Correlation between factors (*p < 0.05)

	Survival	Bone resorption of 1 mm or more within 1 year	Implant placement method
Occurrence of complications	*(*R* = 0.545, *p* = 0.01)	*(*R* = 0.447, *p* = 0.01)	*(*R* = 0.26, *p* = 0.02)
Bone resorption of 1 mm or more within 1 year	* (*R* = 0.53, *p* = 0.02)	–	–
Initial stability less than 60	–	* (*R* = 0.274, *p* = 0.01)	–
Presence of bone grafting	–	–	–
Implant installation method	–	* (*R* = 0.251, *p* = 0.014)	–

*R* correlation coefficient, *p p* value

## Discussion

The stability of the implant is determined by many factors such as the shape of the implant, surface roughness, and surface treatment, among which the surface treatment of the implant is one of the main factors affecting the prognosis of the implant. The surface treatment of the implant aid in increasing the contact between the bone and the implant by improving the wettability of the implant surface, thus improving the osseointegration process [9]. In the SLA surface treatment method, TiO_2_ or Al_2_O_3_ particles are mainly used for surface wear, and many authors report that 75 μm aluminum particles are effective for sandblasting. After the first step of forming such macro-roughness, the second step of forming micro-roughness through acid etching occurs. In most cases, the acid-etching process is carried out with HCl or H_2_SO_4_ solution.

Several studies have reported that the clinical results of implants with SLA surfaces are very good. According to a Hak-Kyun Kim et al. study, a total of 176 implants treated with SLA exhibited a high survival rate of 98.1% in the maxilla and 94.3% in the mandible [5]. Elkhaweldi et al. reported that the survival rate of RBM surface-treated implants was 95.2%, but the survival rate of SLA surface-treated implants was higher than 99.1% [10]. Using this SLA surface treatment method, Osstem (Busan, Korea) developed implants with SA surfaces that were subjected to sandblasting and acid etching using alumina, but more long-term observational studies on the implants are necessary [11–13].

In this study, implant stability was classified into ISQ 60 and above and ISQ 60 and below. The reason for this is based on the results of Rodrigo et al. where it was reported that implant failure rarely occurs when the ISQ measurement is above 60, but that the failure rate is approximately 19% when the ISQ measurement is below 60 [14]. Of the five implants that failed to survive in this study, two implants involved primary stability levels that were less than 60. Factors affecting the primary stability of implants include bone quantity and bone quality. Factors affecting secondary stability include primary stability and bone regeneration ability [15]. Although the study did not accurately examine bone quality and bone mass, it was possible to estimate the possibility of increased marginal bone uptake when the primary stability of the implant was low.

The absorption of marginal bone represents the destruction of marginal bone tissue, which involves bone tissue loss performed by osteoclasts and monocytes [16]. Oh et al. reported that factors affecting early implant marginal bone absorption included surgical trauma, occlusal overload, peri-implantitis, micro-gaps, biological width invasion, and implant platform types [17]. The results of this study showed no significant correlations between marginal bone absorption, implant diameter and length, and bone grafting, but marginal bone resorption was significantly correlated with complications, initial stability, and the implant placement method. When complications occurred, the initial stability was less than 60, and when the implant involved a 2-stage process, the absorption of marginal bone was significantly increased.

Several studies have reported that infections and inflammatory responses following plaque accumulation are associated with progressive marginal bone resorption around implants [18–20]. Mombelli et al. defined peri-implantitis as a site-specific infection, and reported that the microorganisms involved in peri-implantitis were similar to those found in chronic periodontitis [21]. The study also showed increased marginal bone uptake in complications such as peri-implantitis.

Rasoul et al. reported that more marginal bone loss occurred when implants were placed through two-stage surgery than when implants were placed by one-stage surgery [22]. And Hakimeb et al. reported that two-stage implants showed significantly more crestal bone loss than the one-stage implants [23]. Also in this study, more marginal bone absorption occurred when the two-stage method was used for implant placement. The results of the retrospective analysis of the medical records of the study subjects showed that when the initial stability was low, it was placed in a 2-stage process, and when the initial stability was high, it was performed through the 1-stage implantation method. In other words, the lack of bone mass accompanied by bone grafting or poor bone quality is considered to be related to two-stage placement. By comparing the types of implants used, a significant amount of marginal bone resorption was observed in the TS system. This can be due to the fact that in cases of good initial stability, most SS SA system implants were placed in a 1-stage process with the TS SA system being used for most 2-stage cases.

In this study, the causes of the failure of five implants were determined to be due to the failure of initial osseointegration in three cases, infection in one case, and early exposure to the upper part of the fixture in one case. Implant failures are divided into early implant failures during the bone adhesion period and the initial loading period, and delayed implant failures during implant functioning after the bone adhesion process. Early implant failures are due to the disruption of the initial healing process causing the formation of fibrous scar tissue between the surface of the implant and the surrounding bone. The causes include infection or tissue necrosis, trauma during surgery, contamination of the implant, inadequate healing, and excessive loading. In contrast, delayed implant failure is due to a pathological phenomenon in which the biological balance around the implant is disrupted by trauma or infection [24–29]. All cases that failed in this study were associated with early complications.

Early and delayed complications in this study were treated as follows. The infection was resolved by rapid incision and drainage followed by antibiotics after an early diagnosis. Implants that failed due to inadequate initial osseointegration and early exposure of the fixture first underwent treatment involving drug therapy and additional healing time. However, inflammation and mobility continued, and the inflammatory granulation tissue was removed by curettage of the affected area after removal of the fixture. Peri-implantitis could be resolved by peri-implant curettage and flap curettage with respective or regenerative surgery, chlorhexidine irrigation, and local and/or systemic antibiotic treatment. In one case of screw loosening, complications were resolved by screw tightening. In this study, the number of complications, especially prosthetic complications, was very low.

## Conclusion

Of the 96 cases, 5 implants failed resulting in a 94.8% survival rate. The failed implants were all cases of early complications such as infection, failure of initial osseointegration, and early exposure of the fixtures. Peri-implantitis was mostly addressed through conservative and/or surgical treatment and resulted in very low prosthetic complications. Therefore, if preventive measures are taken to minimize initial complications, the results can be very stable.

## Data Availability

The datasets generated and/or analyzed during the current study are available from the corresponding author on reasonable request.

## Abbreviations

SLA: Sandblasted large-grit acid-etched
HA: Hydroxyapatite
ISQ: Implant stability quotients
